# Dietary preferences of brachyuran crabs from Taiwan for marine or terrestrial food sources: evidence based on fatty acid trophic markers

**DOI:** 10.1186/s12983-021-00405-0

**Published:** 2021-05-19

**Authors:** Meike Stumpp, Reinhard Saborowski, Simon Jungblut, Hung-Chang Liu, Wilhelm Hagen

**Affiliations:** 1 University of Bremen, BreMarE (Bremen Marine Ecology), Marine Zoology, PO Box 330 440, 28334 Bremen, Germany; 2 Christian-Albrechts-University, Zoological Institute, Am Botanischen Garten 3-9, 24118 Kiel, Germany; 3 Alfred Wegener Institute, Helmholtz Centre for Polar and Marine Research, Am Handelshafen 12, 27515 Bremerhaven, Germany; 4 Present address: University of Bremen, BreMarE (Bremen Marine Ecology), Marine Botany, PO Box 330 440, 28334 Bremen, Germany; 5 Land Crab Ecology Research Laboratory, 53 Chenggong 11th St., Jubei City, Hsinchu County 302 Taiwan; 6 University of Bremen, MARUM Center of Environmental Sciences, PO Box 330 440, 28334 Bremen, Germany

**Keywords:** Decapoda, Lipids, Triacylglycerols, Fatty acids, Midgut gland, Trophic relationships, Algae, Vascular plants

## Abstract

**Background:**

Trophic interactions are key processes, which determine the ecological function and performance of organisms. Many decapod crustaceans feed on plant material as a source for essential nutrients, e.g. polyunsaturated fatty acids. Strictly herbivorous feeding appears only occasionally in marine decapods but is common in land crabs. To verify food preferences and to establish trophic markers, we studied the lipid and fatty acid composition of the midgut glands of two marine crab species (*Grapsus albolineatus* and *Percnon affine*), one semi-terrestrial species (*Orisarma intermedium*, formerly *Sesarmops intermedius*), and one terrestrial species (*Geothelphusa albogilva*) from Taiwan.

**Results:**

All species showed a wide span of total lipid levels ranging from 4 to 42% of the dry mass (%_DM_) in the marine *P. affine* and from 3 to 25%_DM_ in the terrestrial *G. albogilva*. Triacylglycerols (TAG) were the major storage lipid compound. The fatty acids 16:0, 18:1(n-9), and 20:4(n-6) prevailed in all species. Essential fatty acids such as 20:4(n-6) originated from the diet. Terrestrial species also showed relatively high amounts of 18:2(n-6), which is a trophic marker for vascular plants. The fatty acid compositions of the four species allow to clearly distinguish between marine and terrestrial herbivorous feeding due to significantly different amounts of 16:0, 18:1(n-9), and 18:2(n-6).

**Conclusions:**

Based on the fatty acid composition, marine/terrestrial herbivory indices were defined and compared with regard to their resolution and differentiating capacity. These indices can help to reveal trophic preferences of unexplored species, particularly in habitats of border regions like mangrove intertidal flats and estuaries.

**Supplementary Information:**

The online version contains supplementary material available at 10.1186/s12983-021-00405-0.

## Background

Many decapod crustaceans feed on plant material as a supplementary source of vitamins or essential fatty acids. Exclusively herbivorous feeding, however, is rare in marine species [1], but common in terrestrial species, i.e. land crabs [2, 3]. Some land crabs feed on seeds and seedlings, but most species consume leaf litter, thus, accelerating its decomposition and the turnover of nutrients [2]. In subtropical habitats, up to 79% of plant litter is decomposed by terrestrial crustaceans [4]. Moreover, crabs retain leaves in their burrows, considerably increasing nutrient concentrations in the soil, hence, fulfilling important ecological and biogeochemical functions.

Plant diet is usually of low nutritional quality, i.e. low in nitrogen and lipid levels. Primarily herbivorous crustaceans compensate this by increased feeding rates or by supplementing their diet with fruits or remains of animals [3, 5–7]. A premise for evaluating the impact of species on food webs and nutrient cycling is the knowledge of their feeding preferences and origin of their food. Especially in subtropical habitats and land-sea transition zones, where land crabs are common, it is often unknown, whether a crab species relies mostly on marine or terrestrial food sources. Fatty acid trophic markers can help to identify preferred food sources. The fatty acid trophic marker (FATM) concept is based on the observation that fatty acids (FAs) characteristic of the diet can be detected in higher quantities in the consumer’s lipids [8]. Excess energy gained from the food is stored in body reserves. While the size and lipid content of the midgut gland is an indicator of the crab’s overall condition [9–11], the FA composition of the stored lipids can be used for the identification of food sources. An advantage of this FATM approach is the avoidance of biases, which occur when analyzing, e.g., gut contents. Such analyses tend to overestimate recently ingested food items and to underestimate food items, which are fragile and easy to digest. Hence, gut contents only provide a snapshot impression, whereas FA compositions integrate the feeding preferences of the respective animals over much longer time scales, usually weeks [8, 12, 13]. The FATM concept is well established for pelagic organisms [8, 14], and it has also been applied to benthic organisms [15]. Recently, this concept has been successfully used to compare the feeding preferences of two co-occurring temperate crabs and novel FA-based dietary indices have been established [16]. These indices were developed to specifically detect differences in the consumption of green, brown, and red macroalgae as well as diatoms and animal-based food. In this case, the detected differences in the dietary preference of the invasive Asian shore crab *Hemigrapsus sanguineus* and the native European shore crab *Carcinus maenas* suggested that both species do not compete for food, though living in the same habitat.

**Fig. 1:**
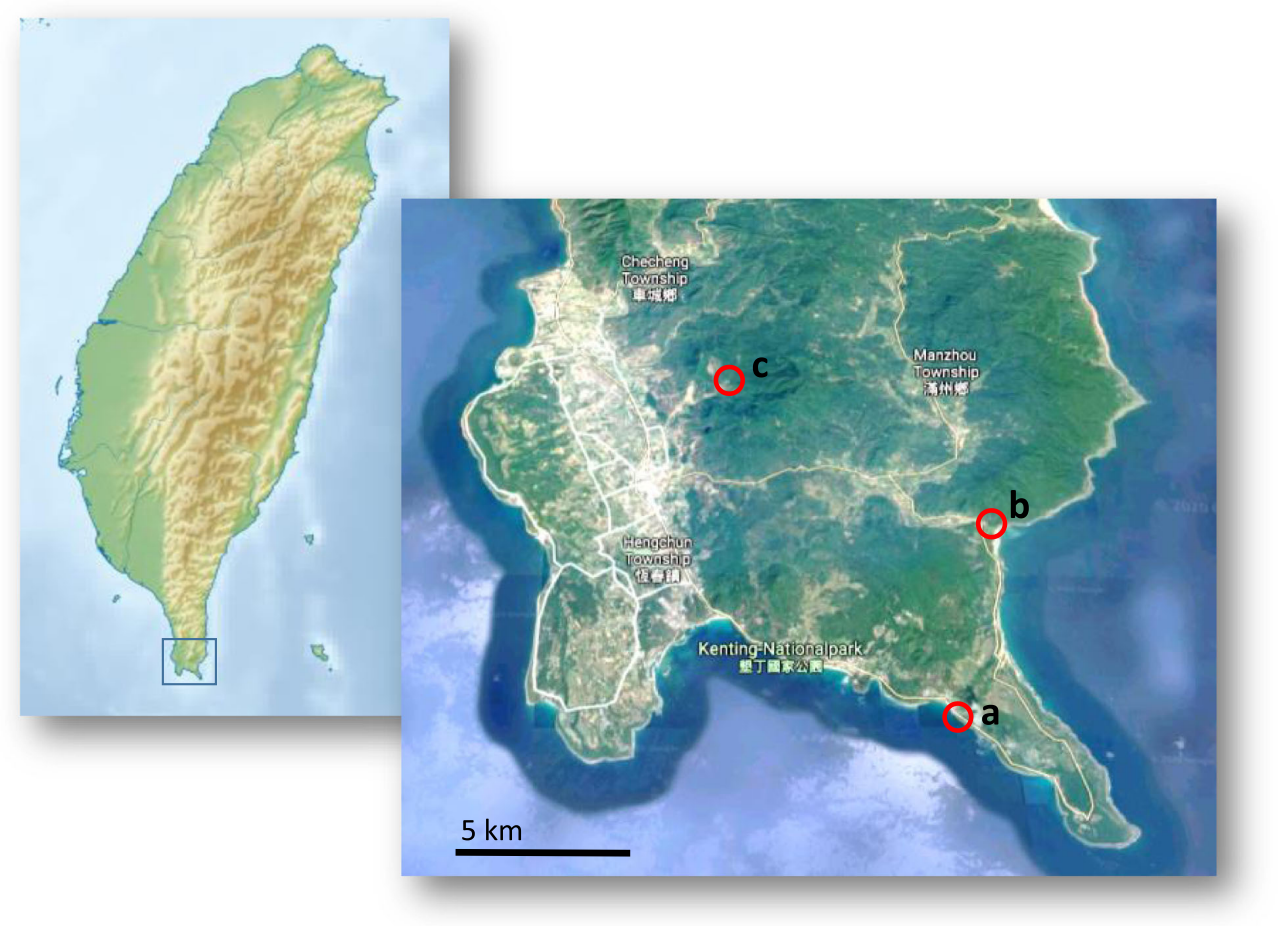
Sampling sites at the southern tip of Taiwan, Hengchun Peninsula. Red circles indicate collection sites of (**a**) *Percnon affine* and *Grapsus albolineatus*, (**b**) *Orisarma intermedium* and (**c**) *Geothelphusa albogilva*. (Sources: Wikimedia.org and google.com/maps, details are given in the Supplementary Information)

To distinguish between marine and terrestrial feeding preferences, we studied the energy storage capacities and FA compositions of four crab species from different habitats in Taiwan (Fig. 1, Table 1): *Percon affine* is a marine species that lives in the lower intertidal of rocky shores down to 10 m depth and it mainly feeds on macroalgae (Hung-Chang Liu, pers. obs.). *Grapsus albolineatus* is a marine species, which can be found above the high tide mark of rocky shores. It feeds primarily on filamentous green algae like *Ulva* spp., but it opportunistically includes carrion in its diet [6, 17]. The semi-terrestrial *Orisarma intermedium* (formerly *Sesarmops intermedius*) occurs along coastal rivers, estuaries, and in salt marshes [18, 19]. It feeds on grass and fresh or old leaf litter [19] (Hung-Chang Liu, pers. obs.). The fully terrestrial *Geothelphusa albogilva* is endemic to Taiwan and occurs along mountain rivers distant from the coast [20]. This species mainly feeds on leaf litter (Hung-Chang Liu, pers. obs.).

**Table 1:** Species names, life styles, and sampling sites of the four crab species (Decapoda, Brachyura) collected in southern Taiwan in May 2007

Species	Family	Life style	Habitat	Feeding mode	Sampling position
*Percnon affine*	Percnidae	marine	lower rocky intertidal	herbivorous	21°55′54.6″N, 120°49′29.0″E
*Grapsus albolineatus*	Grapsidae	marine	spray zone, rock pools	herbi−/omnivorous	21°55′54.6″N, 120°49′29.0″E
*Orisarma intermedium*	Sesarmidae	semi-terrestrial	estuaries, salt marshes	herbivorous	21°59′17.1″N, 120°50′40.2″E
*Geothelphusa albogilva*	Potamidae	terrestrial	mountain rivers	herbivorous	22°02′30.8″N, 120°46′13.3″E

The aim of this study is to develop and test dietary indices according to the FATM concept to determine feeding preferences of the four crab species, and to assess, whether alimentary plant material originated from marine or terrestrial sources. For the development of meaningful indices, we also tested the effect that a low lipid content has on the newly developed indices in the four species.

## Results

A comprehensive data set of this study is presented in the Supplementary Information (S).

### Somatic parameters

**Fig. 2:**
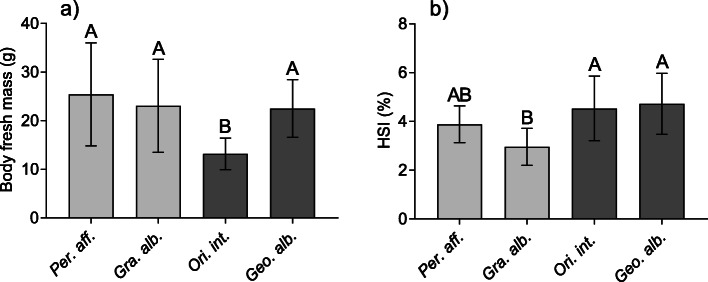
Body fresh mass (**a**) and hepatosomatic index (HSI) (**b**) of the four crab species (light grey: marine species, dark grey: terrestrial species). Means ± SD, *n* = 12. Different letters indicate significant statistical difference (ANOVA, *p* < 0.05)

The marine crabs, *Percnon affine* and *Grapsus albolineatus*, and the terrestrial crab, *Geothelphusa albogilva*, analyzed in this study were of similar size. Their fresh masses were 25.4 ± 10.6 g, 23.1 ± 9.6 g, and 22.5 ± 5.9 g, respectively (Fig. 2a, Table S1). The semi-terrestrial *Orisarma intermedium* was significantly smaller and lighter (13.1 ± 3.2 g) than the other species (one Way ANOVA, F_(3.44)_ = 5.587, *p* = 0.0025).

The hepatosomatic indices (HSI, Fig. 2b, Table S2) were highest in the terrestrial species with 4.5 ± 1.3 in *O. intermedium* and 4.7 ± 1.3 in *G. albogilva*. The HSI of the marine *P. affine* was slightly lower (3.9 ± 0.8). *G. albolineatus* had the lowest HSI (3.0 ± 0.8). The HSI differed significantly between species (one Way ANOVA, F_(3.44)_ = 6.822, *p* = 0.0007).

### Total lipid content

**Fig. 3:**
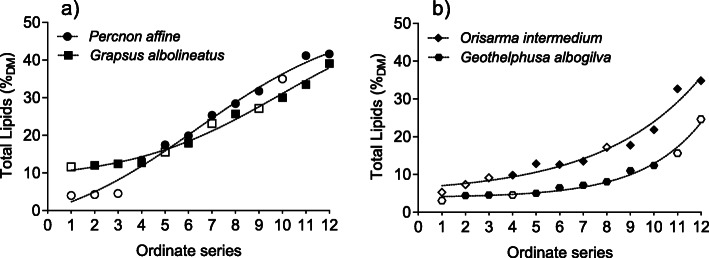
Ordinate series of increasing total lipids in the midgut glands of (**a**) marine species and (**b**) semi-terrestrial and terrestrial species. Filled symbols show samples, which were immediately processed after sampling (*n* = 8 for each species) and open symbols indicate samples after 12 days of starvation (*n* = 4 for each species). The lines are based on a sigmoidal regression model

The total lipid contents (TL in %_DM_) of the midgut glands were displayed on an ordinal scale, ranked from minimum to maximum values (Fig. 3). Lipid levels in the marine species ranged from 4 to 42%_DM_ (average 22.3%) in *P. affine* and from 12 to 39%_DM_ (average 21.7%) in *G. albolineatus*. Data of starved animals, displayed as open symbols, were distributed along the whole scale (Fig. 3a). Applying the sigmoidal model, maximum lipid values were projected to reach about 50%_DM_ in either marine species. The lipid content of the semi-terrestrial *O. intermedium* ranged from 5 to 35%_DM_ (average 16.3%) and that of the terrestrial *G. albogilva* from 3 to 25%_DM_ (average 9.0%) (Fig. 3b). Similar to the other specimens, data of starved animals were distributed along the ascending series. No approximation towards a maximum lipid level could be deduced from the curve.

### Lipid classes

**Fig. 4:**
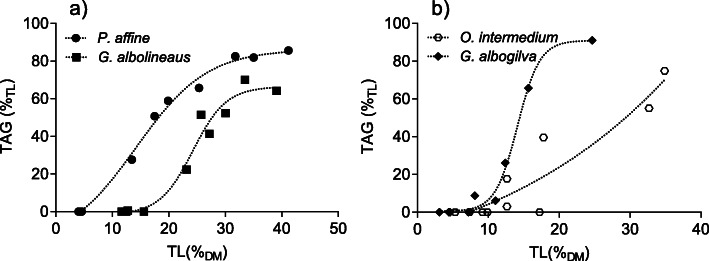
Relationship between total lipids (%_DM_) and the share of triacylglycerols (TAG) in the midgut glands of (**a**) marine species and (**b**) terrestrial species. The dotted lines show the best fit of the sigmoidal regression model (*n* = 12 for each species)

Triacylglycerols (TAG) were the only major storage lipid in all investigated species. The amount of TAG correlated with the total lipid content (Fig. 4).

Maximum values ranged from 70%_TL_ in *Grapsus albolineatus* (Fig. 4a) to 91%_TL_ in *Geothelphusa albogilva* (Fig. 4b). Wax ester and sterol ester (WE/SE) levels were low in all species (< 6.9%_TL_) except in *Grapsus albolineatus*, where WE/SE reached a maximum of 16.6%_TL_. The amount of wax esters showed no statistically significant correlation with the total lipid content. The amounts of free fatty acids (FFA) and sterols (ST) were negligible (Table 2). The shares of polar lipids (PL) were negatively correlated with TAG values.

**Table 2:** Lipid class composition (% of total lipid) of the four crab species. Means ± standard deviation, *n* = 10 for each species (for technical reasons, only 10 of the 12 samples were analyzed)

Lipid class (%_**TL**_)	***Per. aff.***	***Gra. alb.***	***Ori. int.***	***Geo. alb.***
Wax or sterol esters	1.3 ± 0.9	9.2 ± 4.2	1.5 ± 1.5	2.5 ± 2.6
Triacylglycerols	44.8 ± 35.7	30.6 ± 28.8	19.4 ± 28.0	19.9 ± 32.1
Free fatty acids	0.2 ± 0.2	1.2 ± 1.0	0.6 ± 0.6	0.1 ± 0.3
Sterols	3.4 ± 3.4	4.5 ± 2.6	3.2 ± 2.5	6.0 ± 3.0
Polar lipids	50.3 ± 32.5	54.5 ± 27.7	75.2 ± 27.0	71.6 ± 29.2

### Fatty acids and fatty alcohols

The FA compositions of the midgut glands of the four crab species differed from each other (Table 3). Major FA representing more than 10%_TFA_ (%_total fatty acid_), were palmitic acid (16:0), arachidonic acid (20:4(n-6)), oleic acid (18:1(n-9)), and linoleic acid (18:2(n-6)). While 16:0 was the dominant FA in *P. affine* with 27.2%_TFA_, it was lower in *G. albolineatus* (19.0%_TFA_) and *O. intermedium* (13.7%_TFA_). A minimum of 10.8%_TFA_ was present in *G. albogilva*. Percentages of 20:4(n-6) comprised a wide range among marine and terrestrial species: 13.3%_TFA_ and 18.2%_TFA_ for *P. affine* and *G. albolineatus*, respectively, and 13.5%_TFA_ and 18.9%_TFA_ for *O. intermedium* and *G. albogilva*, respectively. 18:1(n-9) was high in the semi-terrestrial *O. intermedium* (16.5%_TFA_) and the terrestrial *G. albogilva* (19.1%_TFA_). In contrast, the 18:1(n-9) values of the two marine species were much lower, accounting for 6.9%_TFA_ in *P. affine* and 9.9%_TFA_ in *G. albolineatus*. The same was true for 18:2(n-6): the marine species *P. affine* and *G. albolineatus* had low levels with 4.1 and 5.9%_TFA_, respectively, whereas *O. intermedium* and *G. albogilva* showed higher values of 10.1 and 11.2%_TFA_, respectively.

**Table 3:** Fatty acid concentrations (percent of total fatty acids, %_TFA_) of the four crab species (mean ± SD, *n* = 12 for each species). Bold numbers emphasize FA contents > 10%_TFA_. “-“ = traces < 0.1%_TFA_ or not detected. * The term “Grasses” comprises species of the genus *Panicum* and *Paspalum*

Fatty acid (%_TFA_)	*Per. aff.*	*Gra. alb.*	*Ori. int.*	*Geo. alb.*	*Ulva* spec.	Grasses*	Leaf litter
14:0	1.6 ± 1.1	0.6 ± 0.7	0.5 ± 0.6	0.1 ± 0.2	2.1	0.6	2.4
16:0	**27.2 ± 9.9**	**19.0 ± 8.6**	**13.7 ± 6.5**	**10.8 ± 3.9**	**27.2**	**19.4**	**26.0**
16:1(n-7)	4.3 ± 2.1	2.6 ± 0.9	2.3 ± 1.6	1.5 ± 1.4	4.0	–	1.5
16:1(n-5)	0.8 ± 0.3	0.4 ± 0.9	1.3 ± 0.4	0.9 ± 0.3	–	–	–
16:2(n-4)	2.0 ± 1.3	1.6 ± 1.0	1.0 ± 1.6	4.2 ± 1.8	0.3	–	0.5
16:4(n-1)	0.1 ± 0.1	–	0.8 ± 0.7	–	–	–	–
17:0	1.6 ± 0.4	1.9 ± 0.5	1.7 ± 0.3	1.4 ± 0.2	1.4	0.7-	1.1
18:0	7.3 ± 4.6	9.6 ± 2.5	8.8 ± 1.9	7.8 ± 1.2	0.8	3.2	**13.5**
18:1(n-9)	6.9 ± 1.5	9.9 ± 1.0	**16.5 ± 5.1**	**19.1 ± 4.3**	5.3	3.6	8.5
18:1(n-7)	3.6 ± 0.7	5.7 ± 1.5	2.0 ± 0.6	1.2 ± 0.4	5.8	0.5	1.1
18:2(n-6)	4.1 ± 0.9	5.9 ± 1.3	**10.1 ± 2.8**	**11.2 ± 3.7**	**14.5**	**15.9**	**12.2**
18:3(n-3)	2.4 ± 0.9	1.9 ± 0.6	2.6 ± 1.3	2.0 ± 1.0	**16.1**	**47.2**	**13.8**
18:4(n-3)	1.5 ± 1.0	0.4 ± 0.6	0.3 ± 1.0	–	3.8	–	–
20:0	0.7 ± 0.3	0.9 ± 0.2	0.8 ± 0.2	1.0 ± 0.2	–	1.6	3.5
20:2(n-6)	1.0 ± 0.4	1.1 ± 0.4	0.9 ± 0.8	1.1 ± 0.5	–	0.3	–
20:3(n-6)	1.2 ± 0.2	1.1 ± 0.2	0.7 ± 0.2	0.9 ± 0.2	0.7	–	0.7
20:4(n-6)	13.3 ± 5.5	**18.2 ± 6.5**	**13.5 ± 7.6**	**18.9 ± 9.3**	2.6	–	0.5
20:5(n-3)	9.8 ± 1.6	5.8 ± 1.8	7.8 ± 3.9	5.3 ± 2.7	2.8	1.3	4.2
22:0	0.5 ± 0.1	0.9 ± 0.4	0.8 ± 0.2	1.2 ± 0.2	0.4	–	–
22:5(n-3)	0.9 ± 0.3	1.2 ± 0.9	0.8 ± 0.2	0.6 ± 0.3	1.5	2.4	3.9
22:6(n-3)	1.6 ± 0.9	1.3 ± 0.9	2.4 ± 0.9	0.9 ± 0.5	0.3	–	–

Among the other FA, palmitoleic acid (16:1(n-7)) was highest in *P. affine* with 4.3%_TFA_, while the other species showed lower values between 1.5 and 2.6%_TFA_. The opposite was detected for stearic acid (18:0): *P. affine* had lowest levels with 7.3%_TFA_ and the other species ranged between 7.8 and 9.6%_TFA_. 20:5(n-3) was relatively high in *P. affine* with 9.8%_TFA_, but only comprised between 5.3%_TFA_ and 7.8%_TFA_ in the other species.

The potential food sources, algae, grasses, and leaf litter, contained high amounts of palmitic acid (16:0) (19.4 to 27.2%_TFA_, Table 3). Leaf litter was also rich in stearic acid (18:0) (13.5%_TFA_). All samples contained high amounts of linoleic acid (18:2(n-6)) and α-linolenic acid (18:3(n-3)), the latter showing a maximum of 47.2%_TFA_ in the grasses.

All species had very low amounts of fatty alcohols, usually 14:0 and/or 16:0. Maximum values of 0.4%_TL_ were present in *G. albogilva*. Apparently, fatty alcohols were minor components in these species and, hence, not considered in further analyses.

**Fig. 5:**
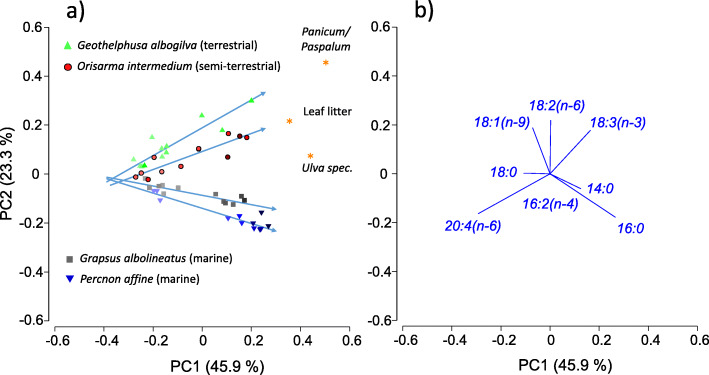
Principal Component Analysis (PCA) of the fatty acid compositions of the four crab species (midgut glands) and three dietary items from Taiwan. Data points (**a**), major vectors (**b**). Increasing lipid content is indicated by increasing intensity of the symbol colors. See also animated gif in the Supplementary Information

The Principal Component Analysis (PCA) of the FA compositions of the four crab species (midgut glands) and the three dietary items revealed a distinct separation of species with increasing lipid content. The first three PCs explained 81.3% of the variation. PC1 covered 45.9% of variation (Fig. 5a, b) and was primarily determined according to their Eigen vectors by the FA 20:4(n-6), 16:0, 18:3(n-3), 14:0, and 18:0 (Tables S3, S4). The individuals of each species lined up according to their lipid content, which was graphically indicated by the intensity of the symbol colors (Fig. 5a). In all crustacean species, the amount of storage lipids correlated positively with the amount of the saturated FA 16:0 and negatively with the FAs 20:4(n-6) and 18:0 (Fig. S1, Table S1). PC2 (23.3%) separated the four crustacean species and particularly the marine species from the semi-terrestrial and terrestrial species. PC2 was primarily determined by 18:2(n-6), 18:1(n-9), and 18:3(n-3). The separation along PC2 was most distinct in the lipid-rich individuals. Lipid-poor individuals of all species showed a more similar lipid composition and approached each other along PC1 and PC2 (Fig. 5a). PC3 covered another 12.1% of the variation and was primarily determined by 18:1(n-9), 18:3(n-3), and 16:2(n-4). It principally separated the crustaceans from the dietary plant material.

### Dietary indices

**Fig. 6:**
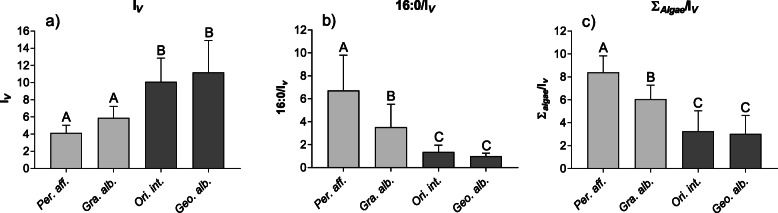
Selected dietary indices are presented: I_*V*_(**a**), 16:0/I_*V*_(**b**), and Σ_algae FA_/I_*V*_(**c**) calculated from the fatty acid compositions of the midgut glands of four crab species from Taiwan. Light grey: marine species; dark grey: terrestrial species. Means ± SD, *n =* 12 each. Bars sharing the same letter are not significantly different from each other (One-Way ANOVA, *p* < 0.05)

Dietary indices varied considerably among the four crab species. The index for the consumption of vascular plants I_*V*_ was lowest in the two marine species *P. affine* (4.1 ± 0.9) and *G. albolineatus*, (5.9 ± 1.4) but significantly higher in the two terrestrial species *O. intermedium* (10.1 ± 2.8) and *G. albogilva* (11.2 ± 3.7) (Fig. 6a, Table S5).

The 16:0/I_V_ index for marine/terrestrial diet decreased significantly from the marine to the terrestrial species (Fig. 6b, Table S5). *P. affine* showed the highest mean value (6.7 ± 3.1), followed by *G. albolineatus* (3.5 ± 2.0), *O. intermedium* (1.4 ± 0.6), and *G. albogilva* (1.0 ± 0.3). The 16:0/I_*V*_ indices of *O. intermedium* and *G. albogilva* were not significantly different from each other.

The second marine/terrestrial diet index, the Σ_algae FA_/I_*V*_ index, showed a similar pattern as the 16:0/I_*V*_ index (Fig. 6c, Table S5). The two marine species, *P. affine* and *G. albolineatus*, had mean values of 8.4 ± 1.5 and 6.1 ± 1.2, respectively. They were significantly different from those of the two terrestrial species, *O. intermedium* (3.3 ± 1.8) and *G. albogilva* (3.0 ± 1.6).

Additional dietary indices of the four crab species are listed in Table 4. The carnivory index I_*Ca*_ of the two terrestrial species, *O. intermedium* and *G. albogilva,* was higher than that of the two marine species, *P. affine* and *G. albolineatus*. The Chlorophyta index I_*Ch*_ was lowest in the terrestrial *G. albogilva*. The index for Phaeophyceae consumption I_*P*_ decreased from the marine towards the terrestrial species, whereas the index for Rhodophyta I_*R*_ was quite similar among the four species. The Bacillariophyceae index I_*B*_ was relatively high in *P. affine*, intermediate in *O. intermedium* and *G. albolineatus*, and low in *G. albogilva*.

**Table 4:** Various dietary indices derived from the midgut gland FAs of four crab species sampled in Taiwan. The dietary indices reflect the consumption of carnivorous diet (I_*Ca*_), Chlorophyta (I_*Ch*_), Phaeophyta (I_*P*_), Rhodophyta (I_*R*_), and Bacillariophyceae (I_*B*_). All values are means ± SD (*n =* 12 for each species)

Dietary index	*Per. aff.*	*Gra. alb.*	*Ori. int.*	*Geo. alb.*
I_Ca_	0.18 ± 0.03	0.25 ± 0.050.	0.47 ± 0.26	0.51 ± 0.22
I_Ch_	6.01 ± 1.24	7.57 ± 1.69	4.56 ± 1.74	3.17 ± 1.12
I_P_	1.48 ± 0.99	0.37 ± 0.64	0.34 ± 0.96	0.00 ± 0.00
I_R_	0.28 ± 0.07	0.21 ± 0.09	0.35 ± 0.21	0.28 ± 0.15
I_B_	4.36 ± 2.16	2.64 ± 0.86	3.11 ± 1.13	1.50 ± 1.38

## Discussion

Information about the feeding preferences and food sources of the brachyuran crabs from Taiwan is sparse and mostly based on anecdotic reports, field observations, feeding experiments, or gut content analyses [17, 21], (Hung-Chang Liu, pers. comm.). Here, we provide lipid and fatty acid profiles, which allow identifying major dietary preferences according to the fatty acid trophic marker concept.

### Energy storage

The hepatosomatic index (HSI) and total lipid levels (TL in %_DM_) of midgut glands are commonly used to characterize the nutritional condition and energy storage capacities of decapod crustaceans. Both HSI and TL of the four crab species ranged within previously reported levels of other (sub-) tropical crabs [22, 23]. The two marine species, *Percnon affine* and *Grapsus albolineatus*, as well as *Orisarma intermedium* showed similar average lipid levels of their midgut glands. Only *Geothelphusa albogilva* had lower TL values, possibly due to its reproductive season, which coincided with the sampling time (Hung-Chang Liu, pers. obs.).

However, individuals of all four species showed a wide range of total lipid levels in their midgut glands ranging from as low as 3 to 4%_DM_ in *G. albogilva* and *P. affine*, respectively, to more than 40%_DM_ in *P. affine* and about 25%_DM_ in *G. albogilva*. This wide lipid range indicates a high individual variability, which seems to differ from the more distinct seasonal lipid cycles of polar zooplankton [24, 25] or crabs and shrimps from higher latitudes [16, 26]. Moreover, low lipid levels were not predominantly assigned to specimens, which starved for 12 days after capture, but were also determined in animals from the field. Some starved animals even exhibited quite high lipid contents suggesting good nourishment before capture.

On average, TL values were lower than those of temperate decapod crabs, which frequently reach 50%_DM_ [10, 16, 27]. Temperature and illumination are constantly high and support a continuous primary production in both, the marine and the terrestrial realm. This leads to a constant food supply of plant material, such as vascular plants or algae. Due to this year-round food availability, tropical and warm temperate marine and terrestrial crabs do not have to accumulate larger energy reserves as for instance required for their cold temperate counterparts from Europe [16, 26], which may explain the generally lower lipd levels. The lipid levels reported here are in line with values of other tropical crabs as, for instance, mud crabs *Scylla paramamosain* from southern China, which reached hepatopancreas lipid levels of about 24.4%_DM_ [28].

Triacylglycerol (TAG) levels were closely correlated with TL levels, as lipids in the midgut glands of decapods are stored predominantly in the form of TAG [26, 29]. TAG as primary storage compound in decapod crustaceans has also been reported for temperate decapod species, namely *Carcinus maenas* and *Hemigrapsus sanguineus* [16], and the pink shrimp *Pandalus montagui* [26]. Correspondingly, the proportion of polar lipids (PL) decreased from low lipid levels towards high lipid levels, as they are rather constant components of total lipids with structural functions, e.g. forming biomembranes.

### Dietary preferences: evidence of FA markers

The high variation in total lipid levels and, thus, in storage TAG allowed detailed analyses of the changes and variations of the FA composition with increasing nutritional status. The individuals of all species with low lipid levels clustered in the PCA, whereas species with increasing lipid contents showed specific allocation patterns. The graphical illustration even suggests that the dispersion of species starts from a common origin and spreads radially with increasing storage lipids. The similarity between the lipid-poor crabs indicates that the basic FA composition, necessary to maintain cell membranes and organ functions, appears similar in all four species. The dissimilarity of the well-fed and lipid-rich animals reflects the different feeding habits and food sources of each species. This makes sense, since dietary fatty acids are accumulated in the depot lipids (i.e., TAG) and the higher the lipid level, the stronger the trophic signatures with a higher resolution [8, 15]. This observation emphasizes the better suitability of the FATM concept for more lipid-rich than for lipid-poor specimens, as shown in many pelagic organisms [8]. It also underlines the need for sufficient sample sizes in lipid and FA studies that represent a wide range of lipid levels to resolve trends and differences in feeding preferences of decapod crabs or other organisms.

All four species show high amounts of arachidonic acid 20:4(n-6), which is, among other polyunsaturated fatty acids (PUFA), considered to be essential and synthesized de novo by plants, e.g., Rhodophyta and Phaeophyta [30]. As marine animals are considered not to be able to synthesize these PUFAs [8], their storage in the midgut glands suggests an at least partially plant-based diet. Thus, the generally high levels of arachidonic acid 20:4(n-6) indicate herbivory, but do not allow to distinguish between terrestrial or marine sources. Overall, the differences in the contributing portions of single FAs still led to separate clusters, or better allocation patterns, of marine and terrestrial species in the PCA.

These differences in FA compositions are also reflected in different dietary indices of the four crab species. However, these indices should not be interpreted independently from each other, as exemplified by the indices for the consumption of algae. The Chlorophyta index is relatively high in the terrestrial *G. albogilva*, which obviously does not encounter chlorophyte algae. The Chlorophyta index is composed of the FAs vaccenic acid 18:1(n-7) and α-linolenic acid 18:3(n-3). The latter FA is also a common component in vascular plants [15] and here it is similarly abundant in all four crab species (Table 4).

The dietary indices of the two terrestrial species are quite similar, although they indicate a higher consumption of diatoms (Bacillariophyceae) by *O. intermedium*. Living along estuaries and in salt marshes, this species may have access to and ingest diatoms, which is unlikely in the case of *G. albogilva*. The dietary indices of the two marine species suggest that *P. affine* feeds on Phaeophyceae to a higher degree than *G. albolineatus*. *P. affine* also seems to ingest more diatoms.

The most striking difference between the two groups of crabs is the strongly differing vascular plant index. This index is almost twice as high in the two terrestrial species compared to the marine species and clearly indicates a higher proportion of a vascular plant diet in the terrestrial species. Thus, this FA trophic marker generally corroborates the sparse knowledge about the herbivorous feeding preferences of these four crab species or close relatives [1, 18, 31–33] (Hung-Chang Liu, pers. obs.).

### Distinguishing marine from terrestrial food sources by FA ratios

16:0/I_*V*_ and Σ_algae FA_/I_*V*_ were defined to differentiate between marine and terrestrial food sources of the crabs. Both indices show the same trends. The ratios decrease the more a crab species is assumed to follow a terrestrial lifestyle. The major difference between the two indices is the inclusion of the membrane FA 16:0. 16:0/I_*V*_ reflects best the gradually increasing importance of terrestrial food sources in the four crab species, from *P. affine* over *G. albolineatus* and *O. intermedium* to *G. albogilva*. However, Σ_algae FA_/I_*V*_ splits the four species more accurately into two groups: *P. affine* and *G. albolineatus* as marine species with significantly higher ratios on the one hand compared to *O. intermedium* and *G. albogilva* as terrestrial species on the other hand.

The applicability of such ratios is supported, when applying the indices to other crab species with a better-known nutritional ecology. The two fully marine brachyurans *Hemigrapsus sanguineus* and *Carcinus maenas* showed high values for the Σ_algae FA_/I_*V*_ ratio of 9.1 and 11.5, respectively, which clearly reflect their marine diet [16]. In contrast, the FA ratios for Σ_algae FA_/I_*V*_ of two semi-terrestrial crayfishes, *Cherax destructor* and *Engaeus sericatus* of 1.3 and 3.2, respectively, indicate a terrestrial diet [34]. However, both pairs of crabs are not strictly herbivorous, similar to the four crab species from Taiwan investigated here. This may explain the differences in the ratios within each pair of crabs, even if *H. sanguineus* and *C. maenas* are both fully marine and *C. destructor* and *E. sericatus* can both be regarded as semi-terrestrial.

## Conclusion

Application and adaptation of the fatty acid trophic marker concept to studies of the feeding ecology and dietary preferences of four decapod crustaceans from Taiwan provide clear evidence that the trophic niches of these largely unknown crab species can be determined. Hence, dietary indices based on trophic marker fatty acids proved to be a suitable tool for identifying food-web relationships. In addition to the already published indices, we propose two new indices for distinguishing crabs having a vascular plant-dominated diet from crabs with marine diets. This may be especially relevant for feeding studies of species living in habitats that are characterized by both food sources, such as mangrove forests and salt marshes.

## Methods

### Origin of samples

Males of the four crab species *Percnon affine* (H. Milne Edwards, 1853), *Grapsus albolineatus* (Latreille in Milbert, 1812), *Orisarma intermedium* (Schubart &Ng, 2020), and *Geothelphusa albogilva* (Shy, Ng & Yu, 1994), were collected in the southern part of Taiwan in May 2007 (Fig. 1, Table 1). Only specimens in the intermoult stage with hardened carapaces were taken. The two marine species were collected during nighttime and transported in aerated seawater to the laboratories of the Providence University in Shalu. The semi-terrestrial and terrestrial species were collected at day and transported in containers with some freshwater to the Shalu laboratories. Additionally, potential food items were collected: *Ulva* spp. for the two marine species, the grasses *Panicum* sp./*Paspalum* sp. for *O. intermedium*, and leaf litter of vascular plants for *G. albogilva*. The collected leaf litter could not be identified to genus or species level. However, the most common plant species around the collection site and, thus, potential terrestrial food sources were *Broussonetia papyrifera*, *Hibiscus taiwanensis*, *Macaranga tanarius*, *Diospyros eriantha*, and *Bischofia javanica*.

Eight individuals per species were immediately processed in the laboratory as described below. To increase the number of specimens with a low lipid content, four individuals of each species were starved for 12 days before tissue samples were taken.

The two marine species, *P. affine* and *G. albolineatus,* were maintained at 25 °C and a light/dark cycle of 12:12 h. *P. affine* was kept in 40 L tanks with aerated seawater. *G. albolineatus* was maintained in tanks (60 × 80 cm), where only the bottom of the tank was covered with seawater. The water was exchanged two to three times per day. The two terrestrial species, *O. intermedium* and *G. albogilva*, were incubated at 25 °C, 80% humidity, and a light/dark cycle of 12:12 h. Freshwater was provided for drinking. Except for *G. albogilva*, all individuals were kept separately in sub-compartments in the tanks to avoid aggression and cannibalism.

### Tissue dissection

The crabs were placed on crunched ice to chill and sedate them. After a few minutes, the crabs were immobile. The fresh mass of each crab was determined and the carapace width was measured. The midgut gland was dissected, the wet mass of the midgut gland was determined, and the tissue was immediately frozen at − 80 °C. The hepatosomatic index (*HSI*) was calculated as the relationship between the fresh mass of the midgut gland (*M*_*MGG*_) and the total crab fresh mass (*M*_*Crab*_):

1 $$ HSI\left(\%\right)=100\ \frac{M_{MGG}}{M_{Crab}} $$

Crab midgut gland samples and plant samples were lyophilized at Shula labs for 48 h (BioTron, Ecospin 3180C) and transported to the Marine Zoology laboratory at the University of Bremen, Germany, on a desiccant (silica gel) and at ambient temperatures of 20–25 °C. After arrival, the samples were immediately stored at − 80 °C.

### Lipid analyses

The dry mass of the lyophilized midgut glands was determined gravimetrically. Subsequently, lipids were extracted with dichloromethane: methanol (2:1, per volume) and an aqueous solution of 0.88% KCl [35, 36]. The mass of the extracted lipids (*M*_*TL*_) was determined gravimetrically and the total lipid (*TL*) content was calculated as percentage of the dry mass of the midgut gland sample (*DM*_*MGG*_):

2 $$ TL\left({\%}_{DM}\right)=100\ \frac{M_{TL}}{DM_{MGG}} $$

Lipid classes were separated and quantified using Thin-Layer Chromatography with an integrated flame ionization detector (MK-5 TLC/FID analyzer, Iatron Laboratories) [37]. Each sample was run in duplicate. Free fatty acid levels were ≤ 1.1%_TL_, indicating that no relevant degradation (autolysis) occurred during sample processing.

Fatty acids (FA) were analyzed after [38, 39]. A subsample of the extracted lipids of each midgut gland was converted to fatty acid methyl esters (FAME) by applying methanol containing 3% concentrated sulfuric acid. The FAME were quantified by gas chromatography (GC). The GC device was equipped with a DB-FFAP column (30 m length, 0.25 mm diameter), a programmable temperature vaporizer injector, and a flame ionization detector (FID). Helium was used as carrier gas. FA were identified by retention times and by using a fish oil standard (Marinol). Free fatty alcohols and most unidentified components accounted for on average less than 1% per species, except for an unidentified component with a mean of 5.5% in *O. intermedium*. Free fatty alcohols and unidentified components were not further considered in the present study.

### Fatty acid trophic markers

The FA dataset was interpreted according to the fatty acid trophic marker concept. The set of dietary indices [16] was adapted and extended to identify food sources of marine and terrestrial origin. In comparison, hexadecatetraenoic acid 16:4(n-3) was removed from the index for carnivorous diet and the Chlorophyta index, because it was not detected in the present samples. Likewise, linoleic acid 18:2(n-6) was omitted from the Chlorophyta index, because it serves as vascular plant index here [8, 15]. Following the species gradient from fully marine to fully terrestrial, the percentage of 16:0 was found to decrease constantly, whereas the opposite was true for the vascular plant index 18:2(n-6) (= I_*V*_; see Results). Thus, the potential of a ratio between these two FA as a marine/terrestrial index was tested. Palmitic acid 16:0 is a biomembrane fatty acid [14, 40]. Therefore, its proportion is also dependent on the amount of stored lipid, i.e. the total lipid content. As a non-biomembrane alternative, a ratio between all algae FA (∑_algae FA_) and 18:2(n-6) was tested as well. All parameters and their equations used in this study are summarized in Table 5. Even though some indices, e.g., algae indices for the terrestrial crabs, are not reasonable per se, we show all results for better comparison. More detailed information on the biomarker function of certain FAs can be found in dedicated review articles [8, 14, 41].

**Table 5:** List of various fatty acid-based trophic marker indices

Carnivory Index	${I}_{Ca}=\frac{18:1\left(n-9\right)}{\left[16:1\left(n-7\right)+16:4\left(n-1\right)+18:1\left(n-7\right)+18:2\left(n-6\right)+18:3\left(n-3\right)+18:4\left(n-3\right)+20:4\left(n-6\right)+20:5\left(n-3\right)\right]}$
Chlorophyta Index	*I*_*Ch*_ = 18:1(*n* − 7) + 18:3(*n* − 3)
Phaeophyceae Index	*I*_*P*_ = 18:4(*n* − 3)
Rhodophyta Index	${I}_R=\frac{20:5\left(n-3\right)}{\left[16:0+18:0+22:6\left(n-3\right)\right]}$
Bacillariophyceae Index	*I*_*B*_ = 16:1(*n* − 7) + 16:4(*n* − 1)
Vascular Plant Index	*I*_*V*_ = 18:2(*n* − 6)
16:0/*I*_*V*_	$16:0/{I}_V=\frac{16:0}{18:2\left(n-6\right)}$
Σ_algae FA_/*I*_*V*_	${I}_T=\frac{\left[16:1\left(n-7\right)+16:4\left(n-1\right)+18:1\left(n-7\right)+18:3\left(n-3\right)+18:4\left(n-3\right)+20:4\left(n-6\right)\right]}{\ 18:2\left(n-6\right)}$

### Statistical analyses

Data sets of HSI and dietary indices were tested for normal distribution with the D’Agostino & Pearson omnibus test. If they passed normality tests, data were compared with a One-Way ANOVA, followed by the Tukey’s post hoc test. If normality tests failed or the number of samples was too small, we applied the Kruskal Wallis test, followed by the Dunn post hoc test. The significance level was α = 0.05.

A sigmoidal model was applied to illustrate the ordinate course of lipid contents as well as the relation between triacylglycerols (TAG) and the total lipid content:

3 $$ f(x)=a+\frac{b}{1+{e}^{\frac{x-m}{s}}} $$

with a = minimum of the fit, b = maximum of the fit, m = point of inflection, 50% level of the function, s = slope at the point of inflection. Statistical analyses and graphical presentation of the data were conducted with the software GraphPad Prism (version 7.05).

A principal component analyses (PCA) was conducted with the entire FA data set of the crabs. FA percentage values of all samples were transformed to proportions (0 to 1) and arcsine-square-root transformed to achieve normality and homogeneity of variances. PCAs were conducted and the corresponding graphs were plotted with the software Primer v7 [42].

## Supplementary Information

**Additional file 1: **Source information of Fig. 1. **Table S1.** Morphometric data of crab species. **Table S2.** Hepatosomatic indices of crab species. **Table S3.** Eigenvalues of PCA. **Table S4.** Eigen vectors of PCA. **Table S5.** Dietary indices complementing Fig. 5. **Fig. S1.** Relation between total lipids and fatty acid contents.

**Additional file 2.** Animated gif. Land crabs PCA.gif.

## Data Availability

The datasets generated during and/or analyzed during the current study will be available in the PANGAEA repository [https://www.pangaea.de].
