# Supplementary material for: Dietary preferences of brachyuran crabs from Taiwan for marine or terrestrial food sources: evidence based on fatty acid trophic markers
Source: Front Zool. 2021 May 19;18:26. doi: 10.1186/s12983-021-00405-0 (PMC8132384; doi:10.1186/s12983-021-00405-0)
Supplement: Supplementary file 1 — Additional file 1: Source information of Fig. 1. Table S1. Morphometric data of crab species. Table S2. Hepatosomatic indices of crab species. Table S3. Eigenvalues of PCA. Table S4. Eigen vectors of PCA. Table S5. Dietary indices complementing Fig. 5. Fig. S1. Relation between total lipids and fatty acid contents. [file 12983_2021_405_MOESM1_ESM.docx]

**Supplementary Information**

Dietary preferences of brachyuran crabs from Taiwan for marine or terrestrial food sources: evidence based on fatty acid trophic markers

**Meike Stumpp^1,2^, Reinhard Saborowski^3^, Simon Jungblut^1,3,4^, Hung-Chang Liu^5^, Wilhelm Hagen^1^**

Figure 1: Source

https://commons.wikimedia.org/wiki/File:Taiwan_relief_location_map.jpg and

https://www.google.com/maps/place/Taipeh,+Taiwan/@21.7743242,120.7414,32545a,35y,36.44t/data=!3m1!1e3!4m5!3m4!1s0x3442ac72bce20a99:0x3f6a35cedd0ac2e0!8m2!3d25.0329694!4d121.5654177)

**Table S1**: Body mass characteristics of crab specimens (n = 12 for each species), SD: standard deviation

**Fresh mass (g) *Per. aff.* *Gra. alb. Ori. int. Geo. alb.***

Min. 12.4 12.4 9.8 14.5

Max. 43.6 38.8 18.8 33.0

Mean 25.4 23.1 13.1 22.5

Median 22.3 20.5 12.2 22.9

SD 10.6 9.6 3.2 5.9

**Table S2**: HSI characteristics of crab specimens (n = 12 for each species), SD: standard deviation

**HSI (%) *Per. aff.* *Gra. alb. Ori. int. Geo. alb.***

Min. 2.75 1.79 2.83 2.26

Max. 5.09 4.52 6.81 6.50

Mean 3.89 2.96 4.53 4.72

Median 3.87 2.80 4.48 4.97

SD 0.75 0.76 1.32 1.25

**Table S3**: Eigen values of principal components.

**PC Eigen values %Variation %Variation (cumulative)**

1 0.0425 45.9 45.9

2 0.0216 23.3 69.3

3 0.0112 12.1 81.3

4 0.0046 5.0 86.3

5 0.0040 4.4 90.7

**Table S4**: Eigen vectors of principal components (coefficients in the linear combinations of variables making up PCs). Grey shades emphasize the most important components.

**Variable** **PC1** **PC2** **PC3** **PC4** **PC5**

14:0 0.252 -0.122 0.081 -0.111 0.049

16:0 0.533 -0.357 0.203 0.092 0.076

16:1(n-7) 0.165 -0.207 0.255 -0.120 0.158

16:1(n-5) -0.103 -0.052 0.056 -0.109 0.085

16:2(n-4) 0.002 -0.010 0.428 0.156 -0.456

17:0 -0.028 -0.044 0.006 0.076 0.092

16:4(n-1) -0.049 0.009 -0.096 -0.429 0.112

18:0 -0.217 0.006 -0.090 0.230 0.499

18:1(n-9) -0.143 0.379 0.572 -0.211 0.179

18:1(n-7) 0.083 -0.245 0.050 0.361 0.171

18:2(n-6) 0.005 0.440 0.050 -0.146 -0.177

18:3(n-3) 0.330 0.357 -0.499 0.121 -0.182

18:4(n-3) 0.175 -0.252 -0.030 -0.325 -0.360

20:0 -0.022 0.074 -0.012 0.119 0.109

20:2(n-6) -0.097 -0.049 0.029 0.072 -0.075

20:3(n-6) -0.008 -0.085 0.090 0.065 -0.024

20:4(n-6) -0.587 -0.328 -0.071 0.198 -0.342

20:5(n-3) -0.137 -0.295 -0.239 -0.471 0.032

22:0 -0.084 0.019 0.131 0.014 -0.031

22:5(n-3) 0.053 0.018 -0.110 0.132 0.162

22:6(n-3) -0.124 -0.106 -0.028 -0.260 0.250

**Table S5:** Dietary indices derived from the midgut gland FAs of the four crab species sampled in Taiwan. The dietary indices reflect the consumption of vascular plants (I*_V_*), and marine/terrestrial diet (16:0/I*_V_* and Σ_algae FA_/I*_V_*). All values are means ± SD of n = 12.

**Dietary index *Per. aff.* *Gra. alb. Ori. int. Geo. alb.***

I*_V_* 4.13 ± 0.90 5.88 ± 1.35 10.09 ± 2.76 11.19 ± 3.71

16:0/I*_V_* 6.73 ± 3.09 3.52 ± 2.00 1.37 ± 0.60 1.00 ± 0.25

Σ*_algae FA_*/I*_V_* 8.40 ± 1.45 6.06 ± 1.23 3.26 ± 1.79 3.02 ± 1.61

Figure S1: Relation between total lipids and fatty acid content of most relevant fatty acids of the midgut glands of the four crab species from Taiwan.

Table S6: Equation parameters of the relation between fatty acid content (% of total fatty acids) and total lipid content according to a one phase association model as shown below.

**Species** **Fatty acid** ***y_min_*** ***y_max_*** ***k*** ***r^2^***

*Percnon affinis* 14:0 -0.8875 2.907 0.06036 0.8673

16:0 -1.676 35.75 0.1019 0.9828

16:2(n-4) -16.49 2.457 0,5672 0.3746

18:0 22.24 3.769 0.1246 0.9893

18:1(n-9) 146.7 6.246 0.9559 0.5348

18:2(n-6) 5.338 3.565 0.06706 0.3033

18:3(n-3) 1.287 142.5 0.00036 0.5911 *1

20:4(n-6) 30.24 9.005 0.1163 0.9687

*Grapsus albolineatus* 14:0 -1.015 7.206 0.01049 0.9054

16:0 -13.16 36.96 0.05192 0.8133

16:2(n-4) -4.123 2,568 0.1076 0.4997

18:0 14.65 -2265 0.00010 0.7834 *2

18:1(n-9) -0.4701 10.05 0.2503 0.0488

18:2(n-6) 2192661 5.538 1.202 0.2022

18:3(n-3) 0.9674 1459 0.00003 0.4521 *3

20:4(n-6) 43.81 4.318 0.05295 0.8592

*Orisarma intermedium* 14:0 -0.8203 1,656 0.0556 0.527

16:0 -2.687 27,52 0.05428 0.7495

16:2(n-4) -1.231 1,359 0.158 0.3463

18:0 11.0 -2.417 0.01146 0.3675

18:1(n-9) 5.689 29.44 0.04126 0.6808

18:2(n-6) 4.549 10.47 0.2452 0.03084

18:3(n-3) -1.196 3.349 0.143 0.2454

20:4(n-6) 33.37 0.1838 0.06473 0.6708

*Geothelphusa albogilva* 14:0 -------- -------- --------- 0.2202 *4

16:0 4.819 39,18 0.02248 0.8443

16:2(n-4) -11.53 5,295 0.487 0.4061

18:0 9.341 -6.185 0.01196 0.652

18:1(n-9) 12.62 39.73 0.03259 0.722

18:2(n-6) -21969 11.58 2.754 0.1044

18:3(n-3) 1.031 1387 0.00008 0.453 *5

20:4(n-6) 34.02 -24.04 0.03594 0.7881

*1 ambiguous fit, alternatively linear fit y = 0.05029 x + 1.289.

*2 ambiguous fit, alternatively linear fit y = 0.2315 x + 14.64.

*3 ambiguous fit, alternatively linear fit y = 0.04175 x + 0.9676.

*4 not converged, alternatively linear fit y = 0.01091 x + 0.03572.

*5 ambiguous fit, alternatively linear fit y = 0.1065 x + 1.031.

-/-
