# Supplementary figures and images for: Dietary preferences of brachyuran crabs from Taiwan for marine or terrestrial food sources: evidence based on fatty acid trophic markers
Source: Front Zool. 2021 May 19;18:26. doi: 10.1186/s12983-021-00405-0 (PMC8132384; doi:10.1186/s12983-021-00405-0)

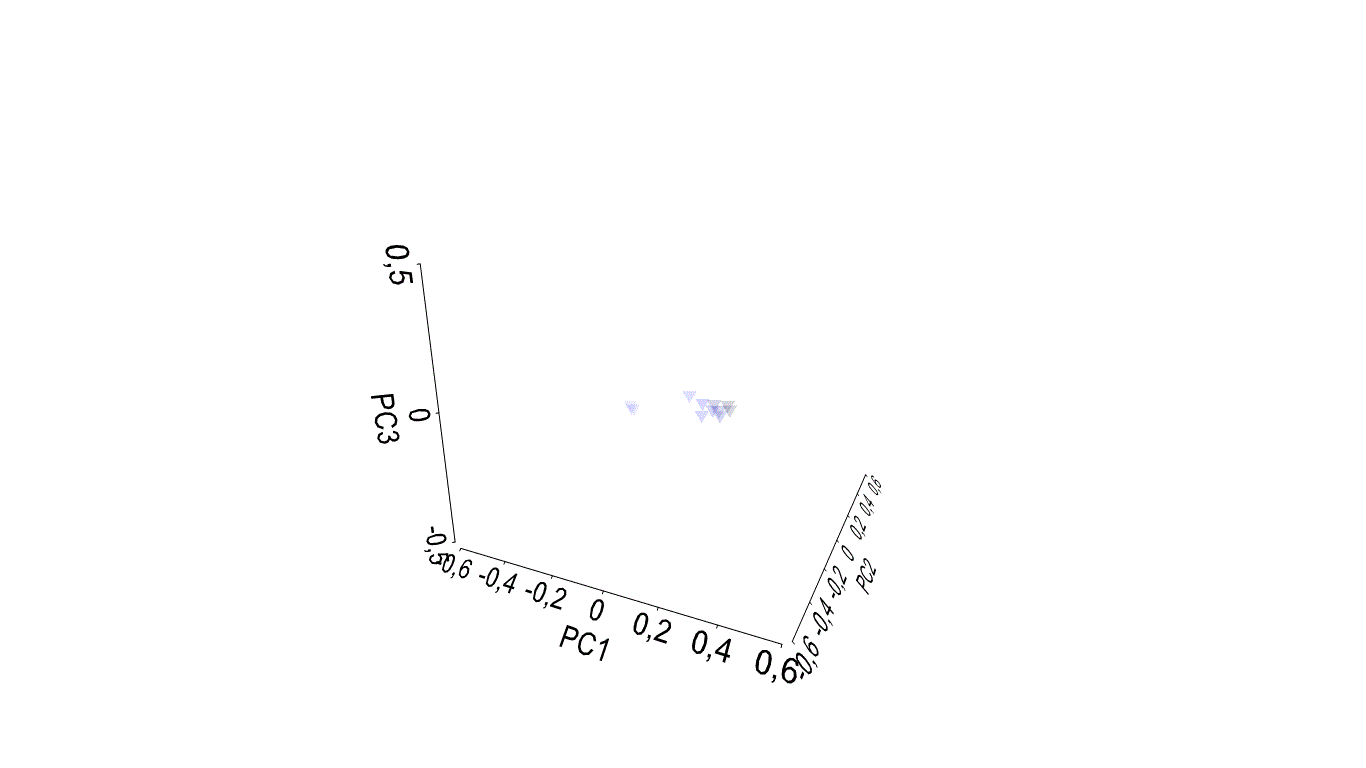

Supplement: Supplementary file 2 — Additional file 2. Animated gif. Land crabs PCA.gif. [file 12983_2021_405_MOESM2_ESM.gif]
